# Alterations of *NURR1* and Cytokines in the Peripheral Blood Mononuclear Cells: Combined Biomarkers for Parkinson’s Disease

**DOI:** 10.3389/fnagi.2018.00392

**Published:** 2018-11-29

**Authors:** Tianbai Li, Zhaofei Yang, Song Li, Cheng Cheng, Bairong Shen, Weidong Le

**Affiliations:** ^1^Center for Clinical Research on Neurological Diseases, The First Affiliated Hospital, Dalian Medical University, Dalian, China; ^2^Liaoning Provincial Key Laboratory for Research on the Pathogenic Mechanisms of Neurological Diseases, The First Affiliated Hospital, Dalian Medical University, Dalian, China; ^3^Institute for Systems Genetics, West China Hospital, Sichuan University, Chengdu, China

**Keywords:** *NURR1*, Parkinson’s disease, biomarkers, cytokines, neuroinflammation

## Abstract

Nuclear receptor related 1 protein (*NURR1*), a transcription factor as key player for maintaining dopamine neuron functions and regulating neuroinflammation in the central nerves system, is a potential susceptibility gene for Parkinson’s disease (PD). To ascertain whether the expression levels of *NURR1* gene and inflammatory cytokines are altered in patients with PD, we measured their mRNA levels in the peripheral blood mononuclear cells (PBMCs) in 312 PD patients, 318 healthy controls (HC), and 332 non-PD neurological disease controls (NDCs) by quantitative real-time PCR. Our data showed that *NURR1* gene expression was significantly decreased in the PBMCs of PD as compared with that of HC and NDC (*p* < 0.01). Since *NURR1* was reported to have regulating effects on neuroinflammation, we assessed the expression levels of cytokines (*TNF*-α, *IL-1*β, *IL-4*, *IL-6*, and *IL-10*) in the PBMCs of PD and controls (HC and NDC). Our results showed that the expression levels of those cytokines were significantly higher than those of controls. Statistical analysis revealed that *NURR1* expression presented a negative correlation with the expression of *TNF*-α, *IL-1*β, *IL-6*, and *IL-10*, and collectively the measurements of *NURR1* plus those cytokines significantly improve the diagnostic accuracy. All these findings suggested that *NURR1* is likely to be involved in the process of PD by mediating the neuroinflammation, and the combination of *NURR1* and cytokines assessment in the PBMCs can be potential biomarkers for PD diagnosis.

## Introduction

Parkinson’s disease (PD) is a chronic and progressive neurodegenerative disorder occurring as a result of the loss of dopamine (DA) neurons within the substantia nigra (SN) (Dickson, 2012). As the second most common neurodegenerative disorder, PD affects 1% of people older than age 60, and 3% at the age 80 years or older (Erkkinen et al., 2018). Although many important discoveries have been made during the 200 years of PD research history (Li and Le, 2017), PD diagnosis is still mainly based on the identification of motor symptoms whereas pathologicalchanges and non-motor manifestations emerge years prior to motor symptoms, indicating that earlier diagnosis and treatments are necessary (Van Laar and Jain, 2004). Currently, an increasing number of studies focus on the peripheral biomarkers utilizing biofluids, which may reflect the disease related molecular changes in the brain. Commonly peripheral blood mononuclear cells (PBMCs) have been used to measure specific alteration in DA components, enzyme activities, DA receptors, and transporters in PD (Caronti et al., 2001; Pellicano et al., 2007; Buttarelli et al., 2011), gene expression profile in PBMCs was widely investigated to identify potential biomarkers of PD.

Causal genes for Mendelian-inherited PD have been reported, including *SNCA, PRKN, UCH-L1, PINK1, DJ-1, LRRK2, GBA*, and *VPS35* (Mizuta et al., 2006; Martin et al., 2011; Williams-Gray and Worth, 2016). Among the potential susceptibility genes which may act as molecular biomarkers of PD, we were particular interested in *NURR1*, a transcription factor belonging to the nuclear receptor 4 family (Wang et al., 2003; Huang et al., 2004). *NURR1* is not only highly expressed in midbrain DA neurons (Castillo et al., 1998; Sakurada et al., 1999), but also in other tissues, such as PBMCs (Mages et al., 1994; Jankovic et al., 2005). *NURR1* is known to play a crucial role in the development and differentiation of midbrain DA neurons (Jankovic et al., 2005; Decressac et al., 2013; Dong et al., 2016). Our previously study has shown that *Nurr1*-null mice have selective agenesis of DA neurons in the SN and ventral tegmental area (Le et al., 1999). Several studies also suggested that dysfunction in *NURR1* gene may play a role in PD (Le et al., 2003; Chu et al., 2006). Our earlier study has documented that *NURR1* gene expression is significantly decreased in the PBMCs of PD as compared with healthy control (HC) and neurological disease control (NDC) (Le et al., 2008; Liu et al., 2012). Based on those reports, it is believed that alteration in *NURR1* could be a potential molecular biomarker of PD.

*NURR1* has also been considered to be a part of anti-inflammatory pathway in microglia, which protects DA neurons against inflammation-induced death (Saijo et al., 2009; Decressac et al., 2013). Mounting evidence supports the role of inflammation as a measurable driving force of PD pathology (Deleidi and Gasser, 2013). Neuroinflammation is associated with activated microglia and altered levels of inflammatory mediators in the brain of PD (Heneka et al., 2014). Many researches have revealed that significantly higher levels of inflammatory cytokines, such as tumor necrosis factor (TNF)-α, interleukin (IL)-1β, IL-4, IL-6 are found in the brain and cerebrospinal fluid (CSF) of PD (Mogi et al., 1996; Nagatsu and Sawada, 2005; Sawada et al., 2006). The pathological effects in the brain may have an implication in the peripheral blood, therefore, detecting the levels of inflammatory cytokines in the peripheral blood may be able to evaluate the inflammatory status of PD (Chen et al., 2008; Reale et al., 2009).

In the present study, we recruited 312 patients with diagnosed PD, 318 HC and 332 non-PD NDC, and measured the levels of *NURR1* and inflammatory cytokines (*TNF*-α, *IL-1*β, *IL-4*, *IL-6*, and *IL-10*) mRNA in their PBMCs. The purpose of this study was to determine whether the expression levels of *NURR1* gene and inflammatory cytokines in their PBMCs were specifically altered in PD as compared with HC and NDC in a relatively larger number of Chinese population, and to evaluate the relationship between *NURR1* and cytokines expression levels in the PBMCs, which may provide further evidence that *NURR1* is involved in the process of PD by mediating the neuroinflammation pathway.

## Materials and Methods

### Participants and Blood Sampling

In this study, we collected a total of 962 PBMCs samples: 312 from patients with sporadic PD, 318 from HC, and 332 from patients with various NDC (Table 1). Among 312 PD patients, 82 were of recent-onset without any anti-PD treatment, the other 230 patients were treated with anti-PD medications. NDC consisted of 48 cerebrovascular diseases, 42 Alzheimer disease (AD), 40 epilepsy, 36 peripheral neuropathy, 31 migraine, 24 myasthenia gravis, 22 essential tremor, 21 parkinsonism (including 11 vascular parkinsonism and 10 multiple system atrophy), 21 anxiety/sleep disorders, 17 restless legs syndrome, 12 motor neuron disease, 11 multiple sclerosis, 4 myelopathy, and 3 chorea minor. PD patients were examined and diagnosed by at least two experienced neurologists from the First Affiliated Hospital of Dalian Medical University according to the Movement Disorder Society Clinical Diagnostic Criteria for Parkinson’s disease (Postuma et al., 2015). PD disease severity was assessed by Modified Hoehn – Yahr (H-Y) staging (Goetz et al., 2004). PD patients were excluded if they had any other major neurological, ongoing infectious/autoimmune, or serious metabolic disorders. HC subjects were recruited from the Health Examination Center of the First Affiliated Hospital of Dalian Medical University, showing they did not have any obvious neurological disorders or non-neurological disorders. All subjects (or their caregivers) recruited to our studies provided a written informed consent agreeing to participate the project. This study has been granted ethical approval by the Ethics Committee of the First Affiliated Hospital of Dalian Medical University (approval number: LCKY2014-29).

**Table 1:** Demographic characteristics of subjects enrolled in the present study.

Groups	Number (%)	Gender Male:Female	*P*-value	*P*-value	Age (years) (mean ± SD)	*P*-value	*P*-value
HC	318(33.1)	178:140	Ref		67.61 ± 8.93	Ref	
PD	312(32.4)	176:136	NS	Ref	67.47 ± 9.56	NS	Ref
NDC	332(34.5)	188:144	NS	NS	67.6 ± 10.2	NS	NS

*Chi-square test and Student’s t-test. SD, standard deviation; Ref, reference; NS, not significant; HC, healthy controls; PD, Parkinson’s disease; NDC, neurological disease control.*

Peripheral blood samples were collected by direct venipuncture at the First Affiliated Hospital of Dalian Medical University. Peripheral blood (2 ml) was drawn from cubital vein into ethylene diamine tetra-acetic acid (EDTA) containing blood collection tubes. PBMCs were separated from Human Peripheral Lymphocyte Separation Medium (Haoyang, Tianjin, China) by centrifugation at 450*g* for 20 min at room temperature (20 ± 2°C) no later than 4 h and were stored at −80°C immediately until RNA preparation.

### PBMCs mRNA Extraction and Quantification

Total RNAs from PBMCs were extracted using the mirVana miRNA Isolation Kit (Ambion, Carlsbad, CA, United States). The mRNA levels of *NURR1*, *TNF*-α, *IL-1*β, *IL-4*, *IL-6*, and *IL-10* in PBMCs were also measured by quantitative real-time PCR. PCR was carried out using ABI 7500 fast real-time PCR system (Applied Biosystems, Foster City, CA, United States) in a total volume of 20 μl for each reaction. *GAPDH* gene was used as an internal control. The specific primers targeting PBMCs *NURR1*, *TNF*-α, *IL-1*β, *IL-4*, *IL-6*, and *IL-10* were presented in Table 2. After 94°C for 30 s, the experimental reaction consisted of 40 cycles of 94°C for 5 s and 60°C for 34 s, the target genes as well as *GAPDH* gene was detected by the fluorescent dye SYBR Green I (TransGen, Beijing, China). The value of threshold cycle (Ct) was generated at every cycle during a run. Fluorescent reading from real-time PCR reaction was quantitatively analyzed by determining the difference of Ct (delta Ct) between Ct of the target genes and *GAPDH*, and the target genes expression were determined using the 2^−dCt^ method.

**Table 2:** List of primers used for quantitative real-time PCR assays.

mRNA	Primer sequence
*NURR1*	Forward: 5′-TCCAACGAGGGGCTGTGCG-3′
	Reverse: 5′-CACTGTGCGCTTAAAGAAGC-3′
*TNF*-α	Forward: 5′-GAGCTGAGAGATAACCAGCTGGTG-3′
	Reverse: 5′-CAGATAGATGGGCTCATACCAGGG-3′
*IL-1*β	Forward: 5′-CTCGCCAGTGAAATGATGGCT-3′
	Reverse: 5′-GTCGGAGATTCGTAGCTGGAT-3′
*IL-4*	Forward: 5′-ACAAAGCCCAGAGAGAACACA-3′
	Reverse: 5′-TCCAACGTACTCTGGTTGGC-3′
*IL-6*	Forward: 5′-AGGACTGGAGATGTCTGAGGCTC-3′
	Reverse: 5′-GCGCTTGTGCAGAAGGAGTTC-3′
*IL-10*	Forward: 5′-CGAGATGCCTTCAGCAGAGT-3′
	Reverse: 5′-GGCAACCCAGGTAACCCTTA-3′
*GAPDH*	Forward: 5′-GAA GGT GAA GGT CGG AGT C-3′
	Reverse: 5′-GAA GAT GGT GAT GGG ATT TC-3′

### Statistical Analysis

Quantitative data were expressed as mean ± standard error of mean (SEM). The dichotomous variables were compared by using chi-square test and continuous variables were compared with independent *t*-test. A one-way ANOVA followed by a Tukey-Kramer test as a *post hoc* analysis was performed using the GraphPad Prism software version 7 (GraphPad Inc., San Diego, CA, United States) to evaluate the differences in the mean value of the relative *NURR1* and cytokines expression. Correlations were evaluated using Spearman’s correlation coefficient (R). The correlations were reported at an α level of 0.05. Receiver operating characteristic (ROC) curves and areas under the curves (AUC) were used to evaluate the prediction performance of the potential biomarkers. The other statistical analysis in this research was performed with the SPSS software version 13.0 (SPSS Inc., Chicago, IL, United States). All statistical checks were carried out two-sided and a *p*-value <0.05 was considered as statistical significance.

## Results

### Characteristics of Study Population

All subjects we collected were ethnic Chinese. The demographic characteristics of PD patients and control subjects were summarized in Table 1. No significant difference in both gender and age was found among patients with PD, HC, and NDC.

### *NURR1* Gene Expression in PBMCs of All Three Groups

**FIGURE 1:**
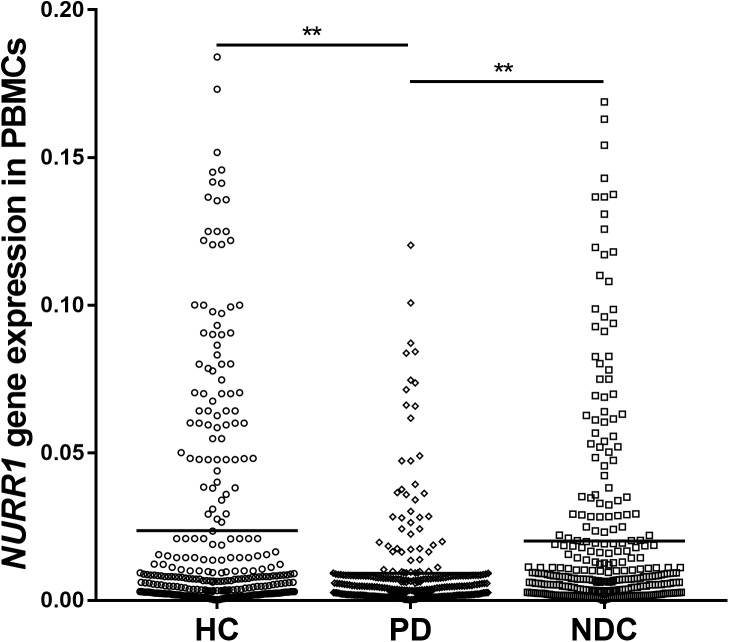
Scatter plots of *NURR1* gene relative mRNA expression level in the PBMCs of HC (*n* = 318), PD (*n* = 312), and NDC (*n* = 332), which was determined using real-time PCR assays. Horizontal bars represent mean value. The *NURR1* gene mRNA level in the PBMCs was markedly low in patients with PD (mean ± SEM, 0.009 ± 0.0009) as compared with HC (mean ± SEM, 0.023 ± 0.0021) and various NDC (mean ± SEM, 0.019 ± 0.002). ^∗∗^*p* < 0.01.

We determined the *NURR1* mRNA level in the PBMCs of all three groups by quantitative real-time PCR technique. We found that the *NURR1* mRNA level in the PBMCs of patients with PD was significantly lower than that of HC (decreased by 61%, *p* < 0.01), and NDC (decreased by 54%, *p* < 0.01). There was no difference of *NURR1* expression between HC and NDC groups (Figure 1). In the individual groups of NDC, some changes were found in the expression levels of NURR1 as compared with PD, but no statistical differences were reached (Supplementary Table S1).

### The Impacts of Disease Duration, Severity, and Medications on *NURR1* Expression in PD

**FIGURE 2:**
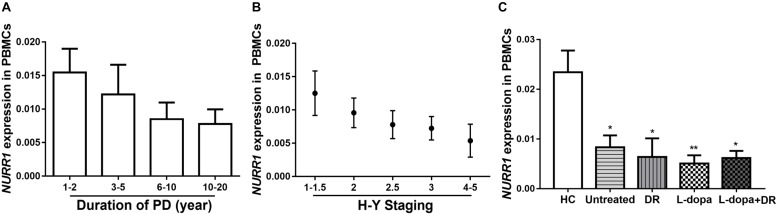
The expression level of *NURR1* in PD and HC. The effects of **(A)** disease course, **(B)** disease severity, and **(C)** medication on the expression level of *NURR1* in PBMCs. The results are the mean ± SEM values. ^∗^*p* < 0.05 and ^∗∗^*p* < 0.01 relative to HC.

We analyzed disease duration (years after onset of disease symptoms) and severity (H-Y scores) in 312 patients with PD. We divided disease duration into four stages: 1–2 years (*n* = 82), 3–5 years (*n* = 83), 6–10 years (*n* = 105), and 10–20 years (*n* = 42) and demonstrated that the level of *NURR1* expression was slightly down-regulated during the disease progression, but no significant statistical difference (Figure 2A). The disease severity of PD was divided into five stages: H-Y 1–1.5 (*n* = 59), H-Y 2 (*n* = 89), H-Y 2.5 (*n* = 93), H-Y 3 (*n* = 55) and H-Y 4–5 (*n* = 16). Again, a slightly down-regulation of *NURR1* expression with higher H-Y scores was found, there was no significant statistical difference (Figure 2B).

We also divided the PD patients into four groups according to the use of medications. Among the 312 PD patients, 82 were untreated with any anti-PD medications, the remaining patients were treated with anti-PD medications, including 89 were treated with l-dopa monotherapy, 28 treated with DA receptor agonists (DR) monotherapy, and other 113 patients were treated with the combination of DA agonists and l-dopa. All these four groups were significantly lower expression level of NURR1 than HC, but there was no significant difference among the four groups (Figure 2C).

### *TNF*-α, *IL-1*β, *IL-4*, *IL-6*, and *IL-10* Expressions in the PBMCs of All Recruited Subjects

**FIGURE 3:**
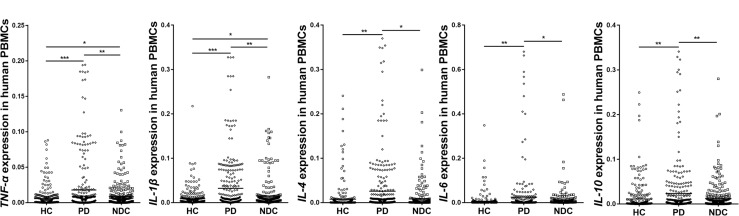
Scatter plots of *TNF*-α, *IL-1*β, *IL-4*, *IL-6*, and *IL-10* relative mRNA expression levels in the PBMCs of HC (*n* = 318), PD (*n* = 312), and NDC (*n* = 332). Significantly higher levels of *TNF*-α (*p* < 0.001), *IL-1*β (*p* < 0.001), *IL-4* (*p* < 0.01), *IL-6* (*p* < 0.05), and *IL-10* (*p* < 0.01) were seen in PD patients than those in HC. The levels of *TNF*-α (*p* < 0.01), *IL-1*β (*p* < 0.01), *IL-4* (*p* < 0.05), *IL-6* (*p* < 0.05), and *IL-10* (*p* < 0.01) expression were also markedly higher in PD than NDC. Horizontal bars represent mean value. ^∗^*p* < 0.05, ^∗∗^*p* < 0.01, and ^∗∗∗^*p* < 0.001.

Since *NURR1* plays important role in regulating neuroinflammation, we then measured mRNA levels of several cytokines in their PBMCs. Our data showed significantly higher levels of *TNF*-α (*p* < 0.001), *IL-1*β (*p* < 0.001), *IL-4* (*p* < 0.01), *IL-6* (*p* < 0.05) and *IL-10* (*p* < 0.05) in PD than those in HC. Moreover, the levels of *TNF*-α (*p* < 0.05), *IL-1*β (*p* < 0.01), *IL-4* (*p* < 0.05), *IL-6* (*p* < 0.05), and *IL-10* (*p* < 0.05) expression were also markedly higher in PD than NDC (Figure 3). In the NDC group, there were slight to moderate differences among different diseases regarding the five cytokines expression levels, but no statistical differences were reached (Supplementary Table S2).

We further analyzed the expression levels of cytokines in different disease duration and severity of PD, no significant differences were found among different groups (Supplementary Figures S1, S2).

### The Influence of Medications on Cytokines Expression

**FIGURE 4:**
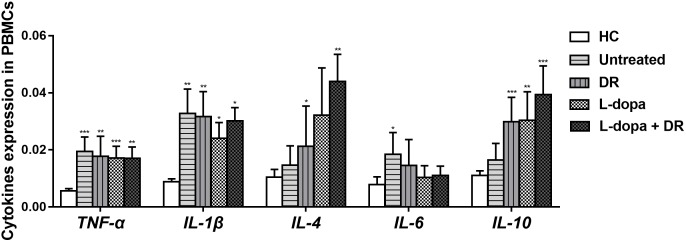
The levels of *TNF*-α, *IL-1*β, *IL-4*, *IL-6*, and *IL-10* expression in PD patients with different anti-PD medications. The results are the mean ± SEM values. ^∗^*p* < 0.05, ^∗∗^*p* < 0.01, and ^∗∗∗^*p* < 0.001 relative to HC. DR: DA receptor agonists monotherapy; L-dopa: l-dopa monotherapy; L-dopa+DR: combination of DA agonists and l-dopa.

Generally, the influence of anti-PD medications on PBMCs cytokines expression was minimal. Our results showed that the levels of *TNF*-α, *IL-1*β, and *IL-6* expression in PD patients with anti-PD medications were slightly lower than that of untreated patients, while the levels of *IL-4* and *IL-10* expression in PD with all types of anti-PD medications were slightly higher than that of untreated PD. However, there was no statistical difference between them (Figure 4).

### Correlations Between the Expression Levels of *NURR1*, *TNF*-α, *IL-1*β, *IL-4*, *IL-6*, and *IL-10*

We performed a correlation analysis between expressions of *NURR1* and *TNF*-α, *IL-1*β, *IL-4*, *IL-6*, and *IL-10.* Our results showed that the level of *NURR1* presented a negative correlation with *TNF*-α (*r* = −0.232, *p* < 0.01), *IL-1*β (*r* = −0.101, *p* < 0.05), *IL-6* (*r* = −0.123, *p* < 0.05) and *IL-10* (*r* = −0.129, *p* < 0.05). Moreover, positive correlations were also found among the expression levels of *TNF*-α, *IL-1*β, *IL-4*, *IL-6*, and *IL-10* (Table 3).

**Table 3:** Spearman correlation coefficient (R) between the expression levels of *NURR1*, *TNF*-α, *IL-1*β, *IL-4*, *IL-6*, and *IL-10* in the PBMCs of PD.

	*TNF*-α	*IL-1*β	*IL-4*	*IL-6*	*IL-10*
*NURR1*	−0.232^∗∗^	−0.101^∗^	−0.058	−0.123^∗^	−0.129^∗^
*TNF*-α		0.372^∗∗^	0.528^∗∗^	0.439^∗∗^	0.337^∗^
*IL-1*β			0.532^∗∗^	0.489^∗∗^	0.411^∗∗^
*IL-4*				0.608^∗∗^	0.462^∗∗^
*IL-6*					0.649^∗∗^

*^∗^p < 0.05; ^∗∗^p < 0.01.*

### Performance of Combined Expression Levels of *NURR1*, *TNF*-α, *IL-1*β, *IL-4*, *IL-6*, and *IL-10* for PD Diagnosis

**FIGURE 5:**
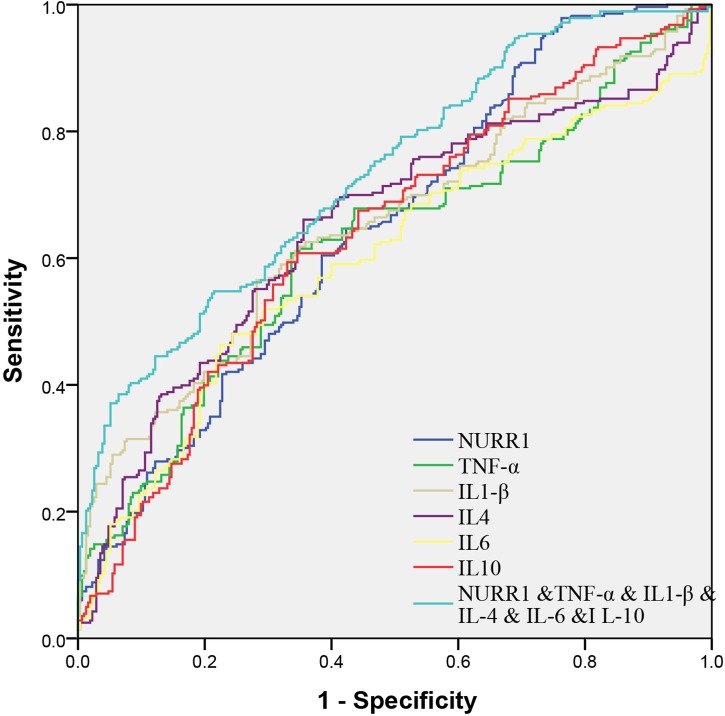
Receiver operating characteristic curves for *NURR1*, *TNF*-α, *IL-1*β, *IL-4*, *IL-6*, and *IL-10* of PD versus HC. The AUCs value of the combination of *NURR1* with cytokines was 0.73 (95% CI, 0.69–0.77; *p* < 0.05), which was out performed those of *NURR1*, *TNF*-α, *IL-1*β, *IL-4*, *IL-6*, and *IL-10* alone.

We evaluated the performance of combined expression of PBMCs *NURR1*, *TNF*-α, *IL-1*β, *IL-4*, *IL-6*, and *IL-10* for the PD diagnosis by the AUC values based on the ROC curve analysis. The AUCs of *NURR1*, *TNF*-α, *IL-1*β, *IL-4*, *IL-6*, and *IL-10* were 0.64 (95% CI, 0.6–0.69), 0.62 (95% CI, 0.58–0.67), 0.65 (95% CI, 0.61–0.7), 0.65 (95% CI, 0.61–0.7), 0.6 (95% CI, 0.55–0.65), 0.64 (95% CI, 0.59–0.68), respectively. The combination of PBMCs *NURR1* with these cytokines significantly enhanced the discriminatory accuracy between PD and HC, with an increased AUC as 0.73 (95% CI, 0.69–0.77) (*p* < 0.05, Figure 5).

## Discussion

In this study, we measured the levels of *NURR1* and inflammatory cytokines (*TNF*-α, *IL-1*β, *IL-4*, *IL-6*, and *IL-10*) mRNA in the PBMCs of a relatively larger number of Chinese population. In order to determine the alterations of PBMCs *NURR1* and cytokines in PD are specific, we particularly recruited 332 various NDC and compared the expression levels of *NURR1* and inflammatory cytokines in PD with not only HC but also NDC. We carried out the assays of five key inflammatory cytokines simultaneously in the PBMCs to assess the complex inflammatory changes in PD. Although there were numerous reports of cytokine changes in PD, a comprehensive analysis of *NURR1* and cytokines expression changes has, to the best of our knowledge, not been performed. We demonstrated for the first time that the level of *NURR1* presented a negative correlation with cytokines in the PBMCs of PD, and the combination of PBMCs *NURR1* and cytokines assessment may improve the diagnostic performance of PD.

Our study confirmed that the expression level of *NURR1* in the PBMCs of PD was significantly lower than that of HC and NDC, which is consistent with our previous reports (Le et al., 2008; Liu et al., 2012). The genetic variant resulting in reduced expression of *NURR1* was reported to be associated with PD (Le et al., 2003; Hering et al., 2004; Jacobsen et al., 2008). *NURR1* has not only been found to be down-regulated in the brains of PD patients (Chu et al., 2006), but also in the peripheral blood of PD patients (Montarolo et al., 2016). These results indicating that *NURR1* dysfunction may contribute to the PD pathogenesis and the disease progression, acting in the brain and in peripheral inflammatory cells.

Based on the evidence that *NURR1* is able to prevent DA neurons from inflammation-induced death through the anti-inflammatory pathway (Bensinger and Tontonoz, 2009; Saijo et al., 2009), we suspected that the decreased level of *NURR1* gene in the pathogenesis of PD may give rise to the expression of inflammatory cytokines. Our study documented that the levels of all measured cytokines were significantly higher in the PBMCs of PD in comparison to the controls. Recently a numerous of studies have reported similar findings in protein levels from samples of serum or plasma (Chen et al., 2008; Hofmann et al., 2009; Reale et al., 2009; Koziorowski et al., 2012). Cytokines are considered key players in the neuroinflammatory cascades associated with the degenerative process in PD (Litteljohn and Hayley, 2012). However, the exact mechanisms by which cytokine levels are elevated in the peripheral blood of PD patients remain controversial. It is believed that neuroinflammation in the central nervous system of PD may induces a systemic inflammatory response to activate mononuclear cells in the peripheral blood to express and produces more cytokines during the disease development and progression of PD (Neumann and Wekerle, 1998; Reale et al., 2009). In the various diseases of NDC, some changes were found in the expression levels of cytokines as compared with PD, but no statistical differences were reached. This could be because of the small number of patients enrolled in the individual groups of NDC. As to the influence of medications on cytokines expression in PD, no statistical difference was found between the anti-PD medications groups and the untreated group, indicating that anti-PD medications may have a minimal effect on the immunological processes of PD.

In this study, we documented that *NURR1* presented a negative correlation with those of *TNF*-α, *IL-1*β, *IL-6*, and *IL-10* in the PBMCs of PD patients. Consistent with our findings, Saijo et al. (2009) showed that *NURR1* acted in microglia and astrocytes to suppress the production of inflammatory mediators and protect against DA neuron degeneration. Another study suggested that the levels of pro-inflammatory cytokines produced by primary microglia was significantly decreased in the presence of *NURR1* overexpression (Chen et al., 2018). These reports suggest that *NURR1* may play a significant role in regulating inflammation in PD. Although statistical analysis showed a negative correlation between *NURR1* and cytokines in our study, it is still unclear how *NURR1* mediates the neuroinflammation in peripheral circulation, and more experiments are needed in the future to clarify that. Furthermore, we also found that the combination of PBMCs *NURR1* and cytokines can enhance the discriminatory accuracy between PD and HC, indicating that combination of *NURR1* and cytokines expression in PBMCs could be utilized as collective biomarkers for PD diagnosis.

## Conclusion

This study may draw the following conclusions: (1) *NURR1* gene expression is significantly decreased in the PBMCs PD patients as compared with HC and NDC. (2) The levels of inflammatory cytokines (*TNF*-α, *IL-1*β, *IL-4*, *IL-6*, and *IL-10*) were significantly higher in the PBMCs of PD patients in comparison to the controls (HC and NDC). (3) *NURR1* presented a negative correlation with *TNF*-α, *IL-1*β, *IL-6*, and *IL-10*. (4) The combination of PBMCs *NURR1* and cytokines assessment could be used as biomarkers and improve the performance of PD diagnosis.

## Author Contributions

TL, ZY, SL, CC, BS, and WL designed the project of this manuscript and revised the paper. TL and ZY carried out all the experiments. TL, ZY, CC, and BS contributed to statistical analyses and results interpretation. TL, ZY, SL, CC, and BS contributed to drafting of the manuscript. WL contributed to research concept and research administration. All authors edited and approved the final version of the manuscript.

## Conflict of Interest Statement

The authors declare that the research was conducted in the absence of any commercial or financial relationships that could be construed as a potential conflict of interest.
