# Supplementary figures and images for: Alterations of NURR1 and Cytokines in the Peripheral Blood Mononuclear Cells: Combined Biomarkers for Parkinson’s Disease
Source: Front Aging Neurosci. 2018 Nov 29;10:392. doi: 10.3389/fnagi.2018.00392 (PMC6281882; doi:10.3389/fnagi.2018.00392)

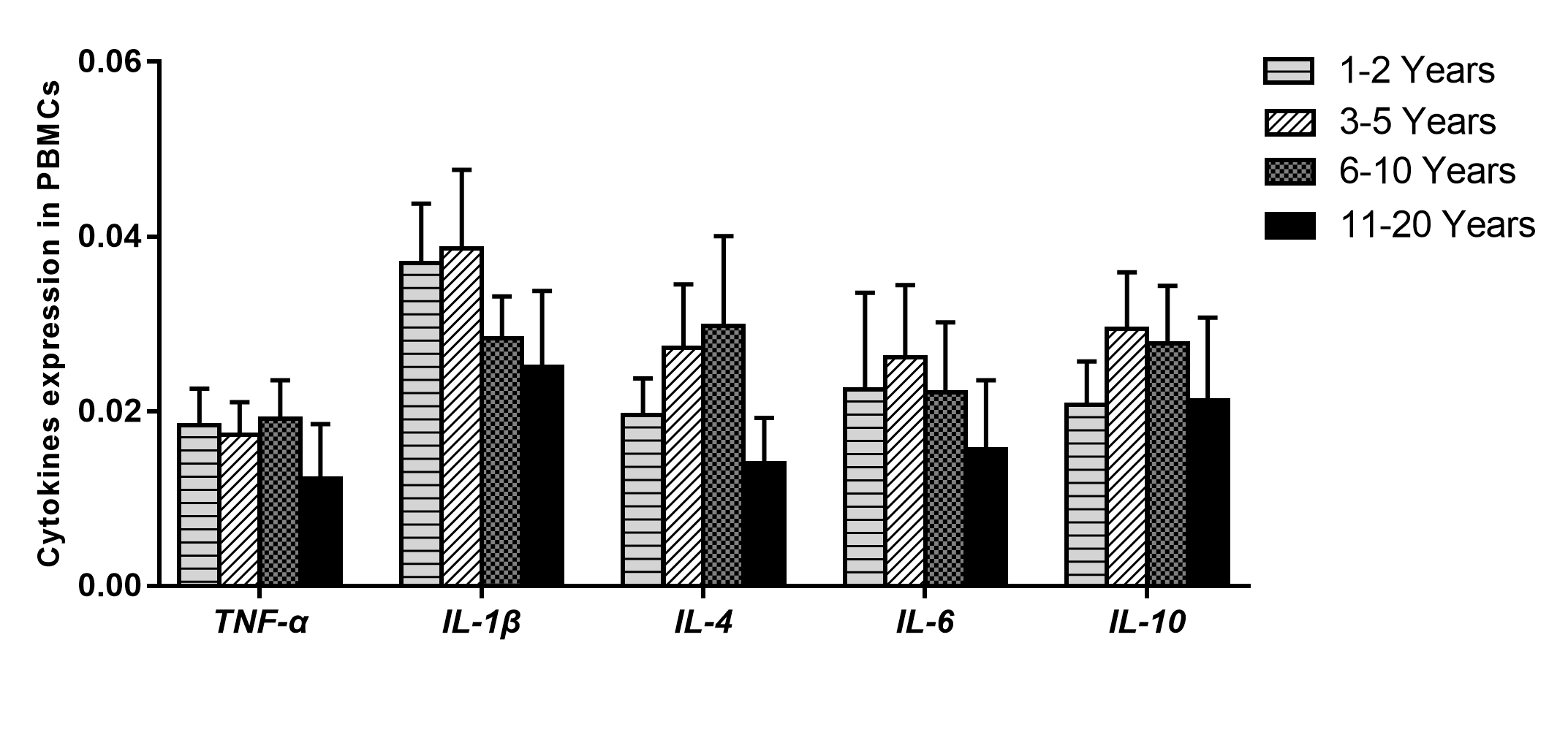

Supplement: Supplementary file 1 [file Image_1.TIF]

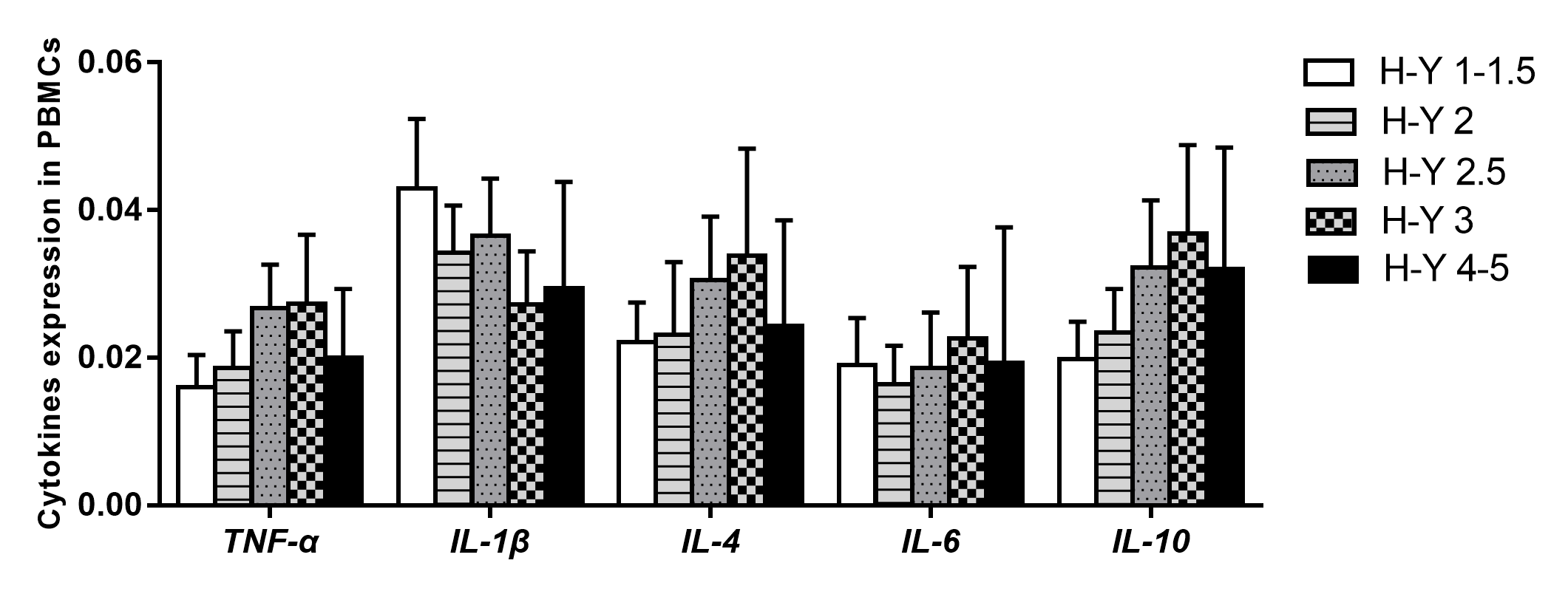

Supplement: Supplementary file 2 [file Image_2.TIF]
