# Supplementary material for: Alterations of NURR1 and Cytokines in the Peripheral Blood Mononuclear Cells: Combined Biomarkers for Parkinson’s Disease
Source: Front Aging Neurosci. 2018 Nov 29;10:392. doi: 10.3389/fnagi.2018.00392 (PMC6281882; doi:10.3389/fnagi.2018.00392)
Supplement: Supplementary file 3 [file Table_1.DOCX]

**Figure Legends**

Supplementary Figure S1. The expression levels of *TNF-α*, *IL-1β*, *IL-4*, *IL-6* and *IL-10* in PD patients with different disease course: 1-2 years (n=82), 3-5 years (n=83), 6-10 years (n=105), and 10-20 years (n=42). The results are the mean ± SEM values.

Supplementary Figure S2. The expression levels of *TNF-α*, *IL-1β*, *IL-4*, *IL-6* and *IL-10* in PD patients with different disease severity: H-Y 1-1.5 (n=59), H-Y 2 (n=89), H-Y 2.5 (n=93), H-Y 3 (n=55) and H-Y 4-5 (n=16). The results are the mean ± SEM values.

**Supplementary Table S1** PBMCs NURR1 expression *vs.* internal control in all groups

| Groups | Numbers | *NURR1* (mean ± SEM) | *P* | *P* |
| --- | --- | --- | --- | --- |
| HC | 318 | 0.023 ± 0.0021 | Ref |  |
| PD | 312 | 0.009 ± 0.0009 | P<0.01 | Ref |
| NDC | 332 | 0.019 ± 0.002 | NS | P<0.01 |
| Non-ND | 268 | 0.021 ± 0.0023 | NS | P<0.05 |
| Cerebrovascular disease | 48 | 0.023 ± 0.006 | NS | NS |
| Epilepsy | 40 | 0.025 ± 0.0063 | NS | NS |
| Peripheral neuropathy | 36 | 0.015 ± 0.0061 | NS | NS |
| Migraine | 31 | 0.024 ± 0.0061 | NS | NS |
| Myasthenia gravis | 24 | 0.02 ± 0.0086 | NS | NS |
| Anxiety/sleep disorders | 22 | 0.023 ± 0.0081 | NS | NS |
| Essential tremor | 22 | 0.022 ± 0.0087 | NS | NS |
| Restless legs syndrome | 17 | 0.018 ± 0.0079 | NS | NS |
| Vascular parkinsonism | 11 | 0.016 ± 0.009 | NS | NS |
| Multiple sclerosis | 11 | 0.019 ± 0.009 | NS | NS |
| ND | 64 | 0.018 ± 0.0049 | NS | P<0.05 |
| AD | 42 | 0.017 ± 0.0068 | NS | NS |
| Motor neuron disease | 12 | 0.022 ± 0.0093 | NS | NS |
| Multiple system atrophy | 10 | 0.018 ± 0.0092 | NS | NS |

Ref=Reference; *P*=*P* Value; NS: not significant; HC: healthy controls; PD: Parkinson’s disease; NDC: neurological disease control; ND: neurodegenerative disease; AD: Alzheimer’s disease; This list displays the disease that numbers in group≥10. *Wilcoxon Rank-Sum (Mann-Whiney Test).

**Supplementary Table S2** PBMCs cytokines expressions *vs.* internal control in all groups

| Groups | Numbers | *TNF-α* (mean ± SEM) | *P* | *P* | *IL-1β* (mean ± SEM) | *P* | *P* | *IL-4*  (mean ± SEM) | *P* | *P* | *IL-6*  (mean ± SEM) | *P* | *P* | *IL-10* (mean ± SEM) | *P* | *P* |
| --- | --- | --- | --- | --- | --- | --- | --- | --- | --- | --- | --- | --- | --- | --- | --- | --- |
| HC | 318 | 0.0057±0.0007 | Ref |  | 0.0089±0.001 | Ref |  | 0.0104±0.0027 | Ref |  | 0.0078±0.0028 | Ref |  | 0.011±0.0017 | Ref |  |
| PD | 312 | 0.0223±0.004 | P<0.001 | Ref | 0.033±0.0036 | P<0.001 | Ref | 0.03376±0.008 | P<0.01 | Ref | 0.0265±0.0074 | P<0.05 | Ref | 0.0267±0.005 | P<0.01 | Ref |
| NDC | 332 | 0.0109±0.002 | P<0.05 | P<0.01 | 0.0203±0.0044 | P<0.05 | P<0.01 | 0.0151±0.0058 | NS | P<0.05 | 0.0125±0.0034 | NS | P<0.05 | 0.012±0.0019 | NS | P<0.01 |
| Cerebrovascular disease | 48 | 0.0127±0.0036 | NS | NS | 0.0193±0.0078 | NS | NS | 0.0179±0.0073 | NS | NS | 0.0098±0.0035 | NS | NS | 0.0136±0.0037 | NS | NS |
| AD | 42 | 0.0066±0.0023 | NS | NS | 0.02±0.009 | NS | NS | 0.0145±0.009 | NS | NS | 0.0147±0.0079 | NS | NS | 0.0146±0.0036 | NS | NS |
| Epilepsy | 40 | 0.0083±0.0022 | NS | NS | 0.0105±0.0041 | NS | NS | 0.0085±0.0053 | NS | NS | 0.0077±0.0033 | NS | NS | 0.0101±0.0037 | NS | NS |
| Peripheral neuropathy | 36 | 0.0143±0.0056 | NS | NS | 0.0095±0.0029 | NS | NS | 0.0138±0.0053 | NS | NS | 0.009±0.0031 | NS | NS | 0.0085±0.0029 | NS | NS |
| Migraine | 31 | 0.0067±0.0029 | NS | NS | 0.0124±0.0065 | NS | NS | 0.0092±0.0047 | NS | NS | 0.0077±0.0043 | NS | NS | 0.0096±0.0038 | NS | NS |
| Myasthenia gravis | 24 | 0.0078±0.0021 | NS | NS | 0.0057±0.0012 | NS | NS | 0.0072±0.0045 | NS | NS | 0.0074±0.0042 | NS | NS | 0.0099±0.0044 | NS | NS |
| Essential tremor | 22 | 0.0082±0.0037 | NS | NS | 0.0125±0.0071 | NS | NS | 0.005±0.0038 | NS | NS | 0.007±0.0025 | NS | NS | 0.0081±0.0034 | NS | NS |
| Parkinsonism | 21 | 0.0124±0.0013 | NS | NS | 0.0218±0.0023 | NS | NS | 0.0251±0.0043 | NS | NS | 0.0132±0.0025 | NS | NS | 0.0157±0.0063 | NS | NS |
| Anxiety/sleep disorders | 21 | 0.0069±0.003 | NS | NS | 0.0106±0.0033 | NS | NS | 0.0059±0.0022 | NS | NS | 0.0085±0.0024 | NS | NS | 0.0084±0.0028 | NS | NS |
| Restless legs syndrome | 17 | 0.0073±0.0046 | NS | NS | 0.0194±0.0067 | NS | NS | 0.0073±0.0039 | NS | NS | 0.0096±0.0037 | NS | NS | 0.011±0.0048 | NS | NS |
| Motor neuron disease | 12 | 0.008±0.0029 | NS | NS | 0.02±0.0127 | NS | NS | 0.0159±0.0068 | NS | NS | 0.0096±0.0057 | NS | NS | 0.0115±0.0049 | NS | NS |
| Multiple sclerosis | 11 | 0.0126±0.0064 | NS | NS | 0.0175±0.0082 | NS | NS | 0.0109±0.0089 | NS | NS | 0.0109±0.0057 | NS | NS | 0.0127±0.0049 | NS | NS |
| Vascular parkinsonism | 11 | 0.0113±0.0051 | NS | NS | 0.0189±0.0072 | NS | NS | 0.0177±0.0064 | NS | NS | 0.0114±0.0074 | NS | NS | 0.0122±0.0032 | NS | NS |
| Multiple system atrophy | 10 | 0.0107±0.0062 | NS | NS | 0.021±0.0047 | NS | NS | 0.0142±0.0043 | NS | NS | 0.0161±0.0052 | NS | NS | 0.0118±0.0045 | NS | NS |

Ref=Reference; *P*=*P* Value; NS: not significant; HC: healthy controls; PD: Parkinson’s disease; NDC: neurological disease control; AD: Alzheimer’s disease; This list displays the disease that numbers in group≥10. *Wilcoxon Rank-Sum (Mann-Whiney Test)
